# Scanning electron microscopy and energy dispersive spectroscopy of Randall’s plaque stones: an unexpected finding of monosodium urate crystals

**DOI:** 10.1007/s00240-025-01842-w

**Published:** 2025-09-13

**Authors:** Victor Hugo Canela, Antonia Costa-Bauzá, Felix Grases, Tarek M. El-Achkar, James E. Lingeman, James C. Williams

**Affiliations:** 1 Department of Anatomy, Cell Biology and Physiology, Indiana University School of Medicine, Indianapolis, IN USA; 2 Department of Pharmaceutical Sciences, College of Pharmacy and Health Sciences, Butler University, 4600 Sunset Ave, Indianapolis, IN 46208 USA; 3 Renal Lithiasis and Pathological Calcification Group, Research Institute of Health Sciences (IUNICS), University of the Balearic Islands, 07122 Palma, Spain; 4 Department of Medicine, Division of Nephrology, Indiana School of Medicine, Indianapolis, IN USA; 5 Department of Urology, Indiana University School of Medicine, Indianapolis, IN USA

**Keywords:** Kidney stones, Randall’s plaque, Monosodium urate, Inflammation, Calculi

## Abstract

Randall’s plaques (RP) are located at the papillary tip, originating in the basement membranes of the thin loops of Henle, vasa recta and collecting ducts, and are associated with kidney stone retention. Disruption of the papillary epithelial layer exposes interstitial RP to calyceal urine, enabling calcium oxalate monohydrate (COM) overgrowth and papillary RP stone formation. This study aimed to analyze the surface and internal structures of RP stones using scanning electron microscopy (SEM) and energy dispersive spectroscopy (EDS). Stones were collected from patients during percutaneous nephrolithotomy, ureteroscopy or both. Eighteen stones from nine patients were examined by stereoscopic microscopy, micro computed tomography (micro CT), SEM and EDS. Seven RP stones were sectioned for internal structure analysis. SEM revealed mineralized tubules potentially originating from thin loops, collecting ducts, ducts of Bellini, or vasa recta. These were frequently covered by collagen fibrils, and some were filled with dense or particulate mineral. Calcium phosphate (CaP) apatite was observed in various crystallized phases within RP regions. In three of the seven sectioned RP stones, monosodium urate monohydrate crystals were intercalated with RP, confirmed by EDS. Our multimodal imaging approach provides new insights into RP composition. This study suggests that sodium urate may precede RP formation in a subset of cases, potentially due to early, unexpected urinary pH shifts. Further studies are needed to validate this hypothesis and advance our understanding of RP stone pathophysiology, informing better diagnostic and therapeutic strategies for kidney stone disease.

**Supplementary Information:**

The online version contains supplementary material available at 10.1007/s00240-025-01842-w.

## Introduction

It is estimated that kidney stone disease affects 10% of Americans, and up to 13% of the global population will experience at least one stone event in their lifetime [1, 2]. As extensively cited in other works, approximately 80% of kidney stone cases are primarily of the calcium oxalate (CaOx) mineral type and display no signs of systemic disease [3, 4].

Idiopathic calcium oxalate monohydrate (COM) papillary stone formers have been previously categorized as at least two distinct phenotypes [5, 6]. One phenotype is characterized by the growth of COM stones on the interstitial mineralization of calcium phosphate (CaP, apatite) known as Randall’s plaque (RP) at the renal papillary tip (Randall et al. 1937, [5, 8]. The other phenotype is characterized by the growth of COM stones on to plugs of apatite crystals in the terminal collecting duct lumens [9, 10]. The mechanisms by which each of these COM stone formers develop nephrolithiasis remains poorly understood.

Insights into the complexities of RP have been highlighted in the literature. For example, Luis Cifuentes Delatte (1987) and Michel Daudon (2007) demonstrated that RP is not always exclusively composed of CaP. Cifuentes et al. examined 142 COM papillary stones and found that 28 of them contained atypical plaques. Among these 28 atypical plaques, 5 contained sulfur (S) and potassium (K), with 3 of these patients confirmed to have been taking sulfonamides for an extended period. Additionally, 2 plaques contained monosodium urate, 1 contained uric acid and 1 contained silicon (Si). These constituents were confirmed using scanning electron microscopy (SEM) and Energy Dispersive X-ray Analysis (EDAX) to determine their atomic composition [11]. Moreover, Daudon et al. reported the relative prevalence of the main components of Randall’s plaques, identifying 3.5% sodium hydrogen urate, 0.5% uric acid and 0.4% other phosphates among the analyzed RP stones [12, 13].

Another important study, which focused on the interface between RP and COM kidney stones, used SEM, X-ray computed tomography (XCT), and energy-dispersive X-ray analysis (EDX) to investigate the morphology and mineral composition of four kidney stones from patients with COM stone-formation [14]. The authors examined the RP-COM interface in one stone, showing that the fibrous structure of calcified tubules influenced the nucleation and growth of COM crystals on to RP. Their findings suggest that RP functions as a porous interface, facilitating ionic diffusion between the plaque and the calyceal urine, ultimately contributing to the crystallization process and stone retention.

In this study, our goal was to analyze RP stones by using stereoscopic microscopy, micro computed tomography (micro CT), SEM, and energy dispersive spectroscopy (EDS) to identify patterns or structural differences that may provide novel insights into the components contributing to RP formation in the renal papilla. By leveraging these combined techniques, we examined the microenvironment of the surface and internal regions of RP stones, aiming to uncover new insights into the pathophysiology of papillary COM stone disease.

## Materials and methods

RP stones were collected from patients during surgery (percutaneous nephrolithotomy, stone removal by ureteroscopy or both) (Supplemental Table 1). A standard protocol for the study of kidney stone patients approved by the Indiana University (IU) Institutional Review Board (IRB) committee (IRB protocol #1,010,002,261) was followed throughout the present study [46]. Additionally, the analyses presented in this study on the RP stone specimens were conducted in accordance with the protocol established by the Laboratory of Renal Lithiasis Research and Biobank of Renal Calculi (BICUIB) of the University of the Balearic Islands.

Dry stones were photographed and scanned using micro-computed tomographic (micro CT) imaging to verify and determine the presence of plaque and stone mineral composition. The stones were scanned using a Skyscan 1172 micro CT system (Bruker-MicroCT, Kontich, Belgium) at 60kVp, 0.5-mm Al filter, for a final voxel size of 3–8 µm [16].

As described in previous publications, kidney stones were sectioned, imaged and captured using stereoscopic microscopy and a scanning electronic microscope (SEM; TM4000 Plus II, Hitachi, Tokyo, Japan) with microanalysis by X-ray dispersive energy (RX, Quantax 75 EDS microanalyzer, Bruker, Berlin, Germany) [17, 18].

## Results

**Fig. 1:**
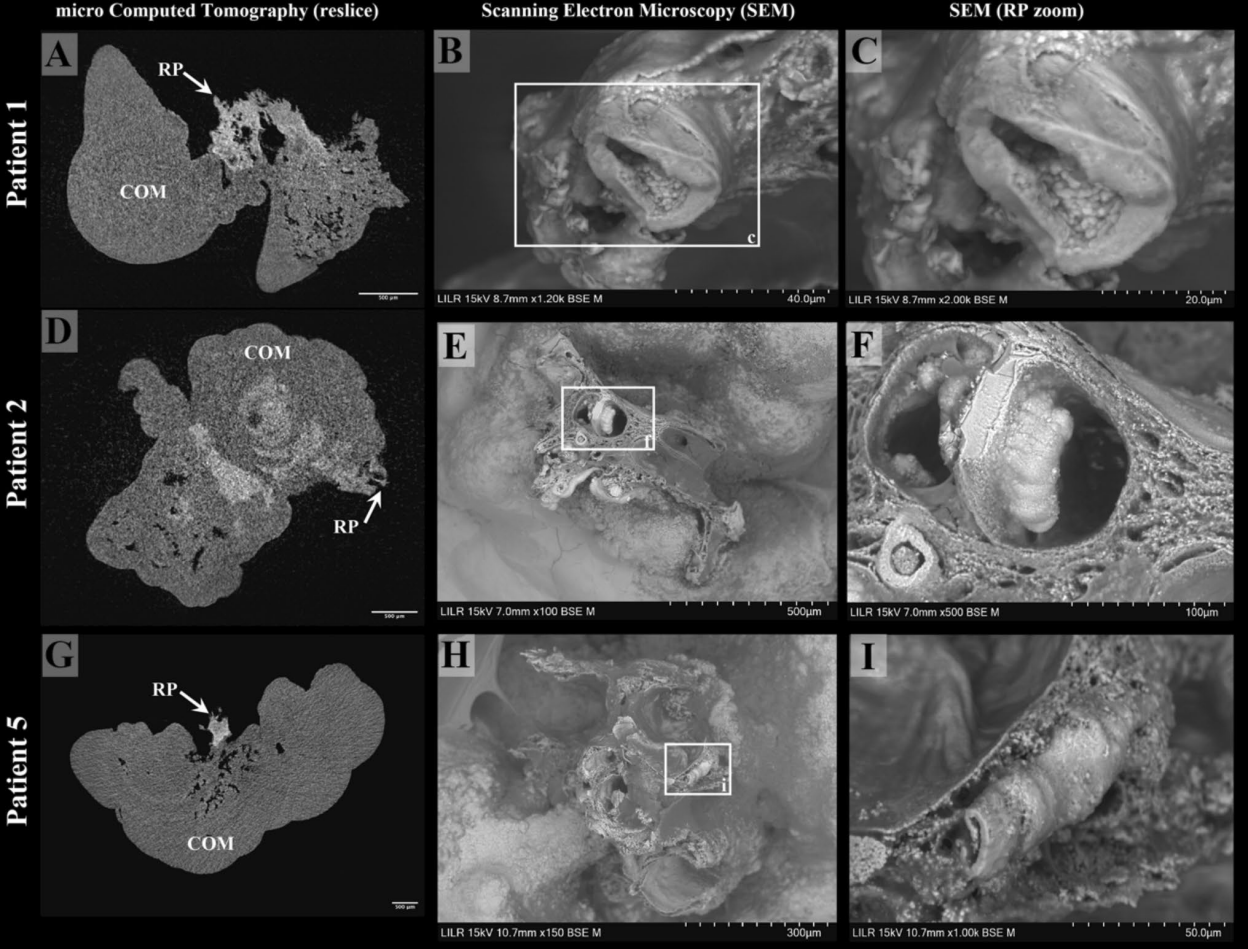
Micro CT and SEM analysis of Randall’s plaque (RP) kidney stones from patients 1, 2, and 5. Micro CT reslice images showing calcium phosphate (CaP, apatite) in RP regions **(A, D, G)**. SEM images reveal mineralized tubules, potentially originating from thin loops, collecting ducts, or ducts of Bellini **(B, E, H)**. Tubules are often covered with collagen fibrils, with dense or particulate mineral deposits (**C** and **I**). Example of an elliptical mineralized tubule (~ 35.18 µm × 21.7 µm), likely from the loop of Henle **(C)**. In Fig. 1F, top views of RP areas in patient 2 show ruptured and dilated tubules (~ 30–35 µm diameter), with some larger dilated tubules (~ 90–110 µm). Patient 5’s RP plaque features broken tubules (26–40 µm) and large papillary duct casts (~ 100–113 µm) (**H** and **I**)

RP stones were analyzed by micro CT, revealing the presence of CaP as shown in Fig. 1 (**A**, **D**, and **G**) for patients 1, 2, and 5, respectively, from the Supplemental Table 1. Figure 1B, C, E, F, H, and I depict SEM imaging of RP kidney stones, revealing mineralized tubules potentially originating from thin loops, collecting ducts, or ducts of Bellini. These tubules were often covered by organic material, likely collagen fibrils, with some containing dense or particulate mineral deposits of apatite. CaP apatite was identified in various crystallized phases within the RP area. Figure 1C illustrates a mineralized tubule with an elliptical shape, measuring approximately 35 µm along its major axis and 22 µm along its minor axis, likely corresponding to a collecting duct. The RP stone from patient 2 features an RP area with multiple tubule structures, some of which are ruptured and/or dilated, while others are either filled or empty. Figure 1E and F highlight tubules measuring 30–35 µm in diameter, that appear to be draining into larger, dilated structures (approximately 90–110 µm) that may represent merging collecting ducts or papillary ducts of Bellini. The RP stone from patient 5 shown in Fig. 1H and I, shows an area of RP with extensive matrix with an area consisting of broken tubules (collecting ducts) of typical size (26 µm-40 µm), and what appear to be casts of ducts of Bellini of significant size (100 µm-113 µm).

**Fig. 2:**
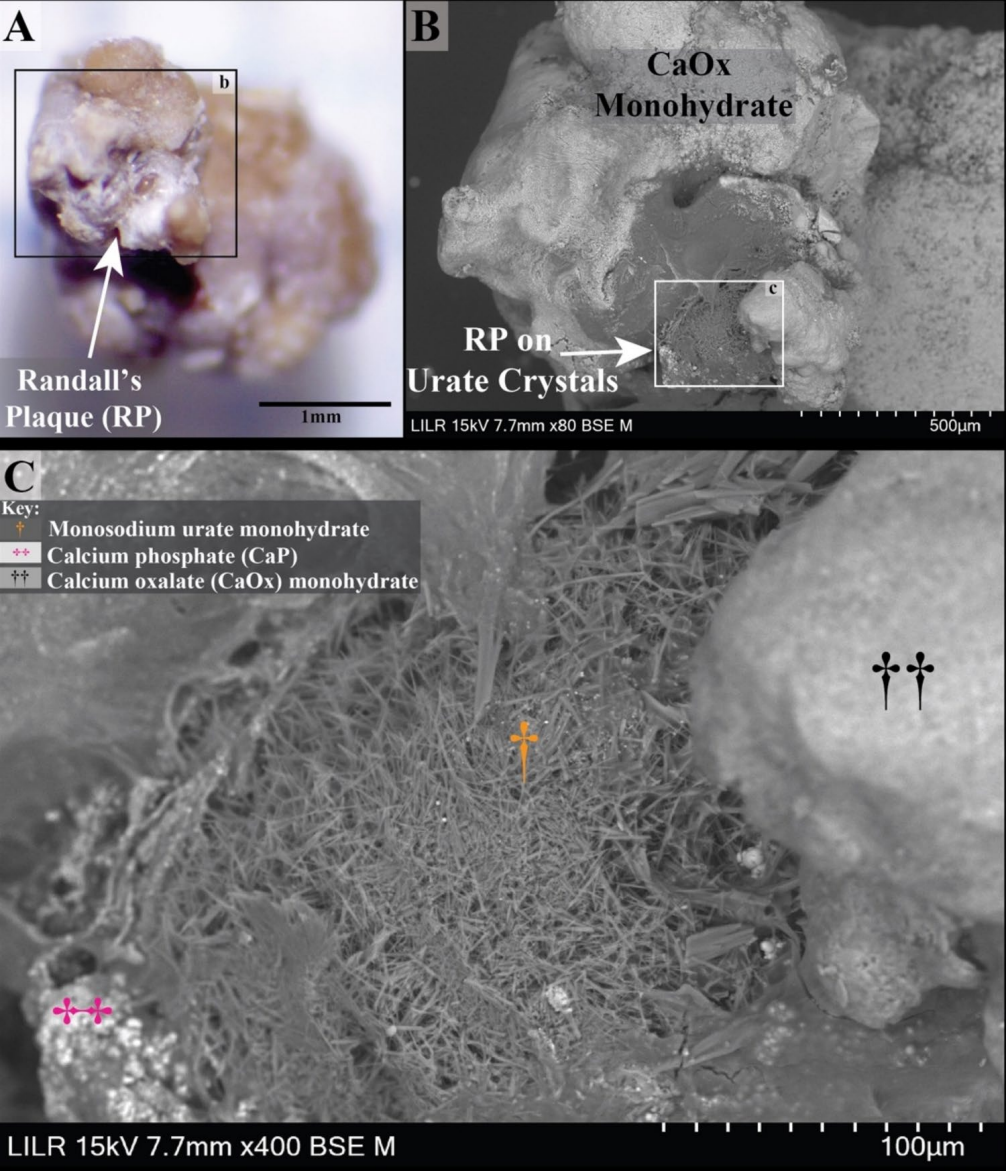
Randall’s plaque (RP) stone from Patient 5, removed via percutaneous nephrolithotomy. **A** Stereoscopic image showing the RP surface for SEM analysis **(B, C)**. SEM images highlighting needle-shaped crystals resembling monosodium urate monohydrate (MSU) within the RP. **C** Close-up view (inset from B) reveals detailed crystal structures, including calcium phosphate (CaP), calcium oxalate monohydrate, and MSU crystals

**Fig. 3:**
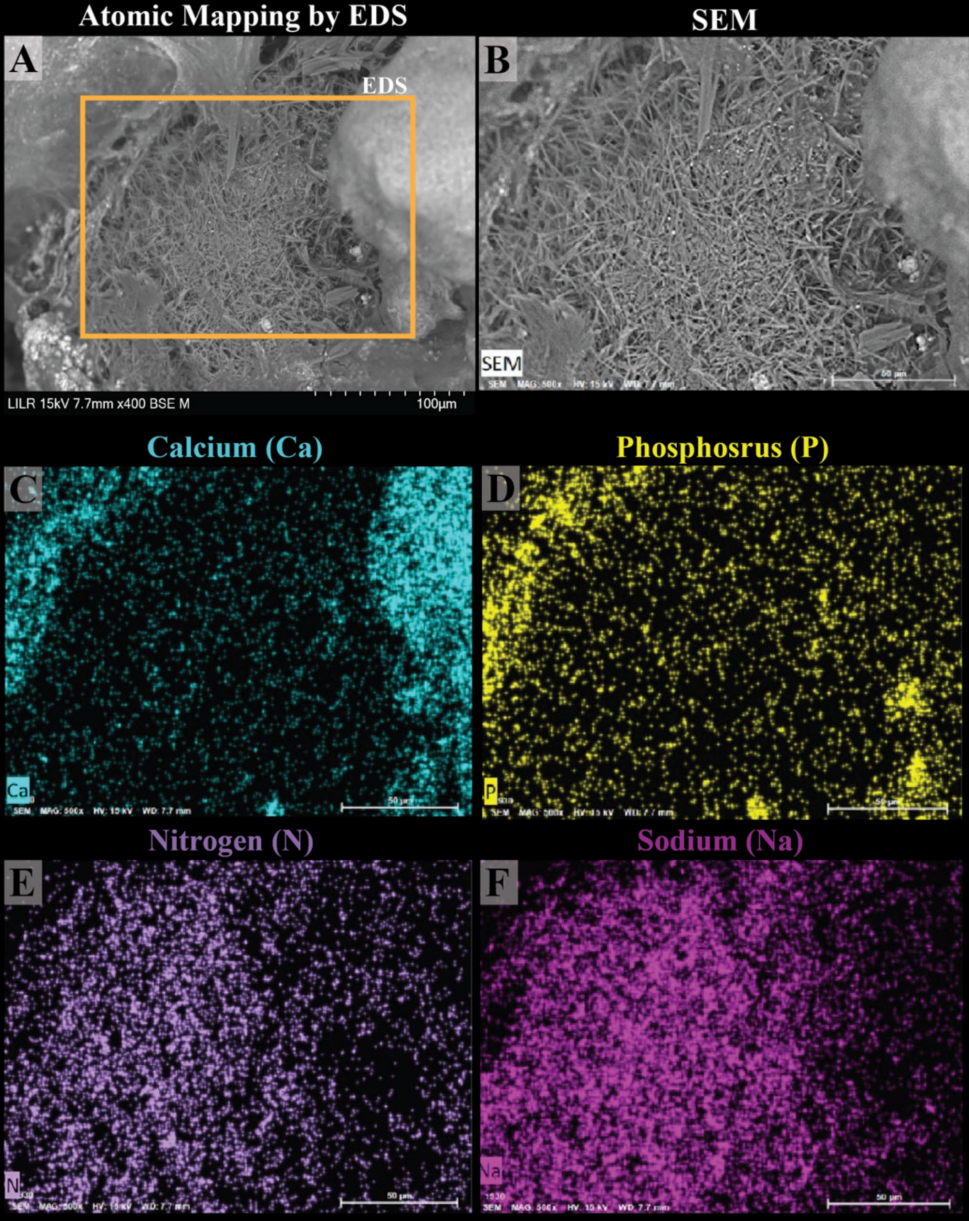
Energy dispersive spectroscopy (EDS) analysis confirms the needle-shaped crystals in Randall’s plaque (RP) as MSU. EDS analysis of the same stone from Fig. 2 confirms the needle-shaped crystals in RP as MSU, with nitrogen **(E)** and sodium **(F)** present in these regions. Areas lacking MSU crystals exhibit apatite (CaP) crystals, typical of RP, as shown in **(D)**

Figure 2 displays one of seven Randall’s plaque stones (stone #4) from patient 5 (Fig. 1G shows micro CT of stone #7). Figure 2A presents a stereoscopic image of the stone, with the surface of Randall’s plaque exposed for detailed SEM analysis. The SEM images in Fig. 2B and C specifically highlight RP containing needle-shaped crystals resembling monosodium urate monohydrate (MSU) crystals. Figure 2C provides a magnified view of the region marked as inset “**c**” in Fig. 2B, showing the detailed structure of calcium phosphate, COM, and MSU crystals. EDS analysis confirms the presence of MSU as needle-shaped crystals as shown in Fig. 3.

Figure 3E and F illustrate the presence of nitrogen and sodium, respectively, in regions where these needle-shaped crystals are located. In contrast, areas without these crystals reveal the presence of apatite crystals (CaP), by the detection of phosphorus in Fig. 3D.

**Fig. 4:**
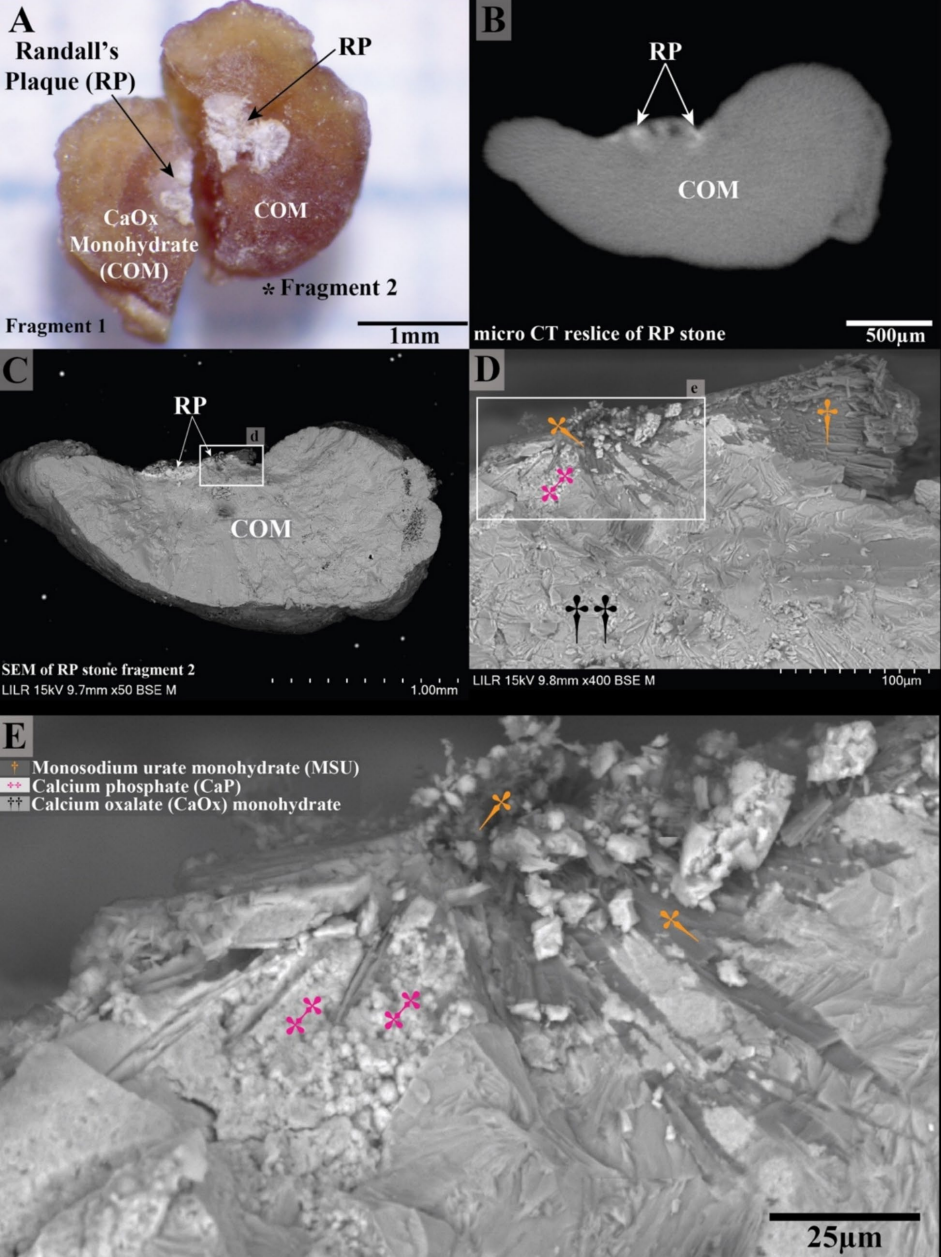
Randall’s plaque (RP) stone from patient 6 shows MSU crystals integrated with apatite crystals within the RP. **(A)** Stereoscopic image showing the RP surface after meticulous sectioning for SEM analysis (**C**, **D** and **E**). **(B)** Micro CT reslice of the stone shows the approximate section viewed by SEM. SEM images emphasize the needle-shaped MSU crystals within the RP. **(E)** A close-up view (inset from D) reveals detailed crystal structures, including calcium phosphate (CaP) and MSU crystals

**Fig. 5:**
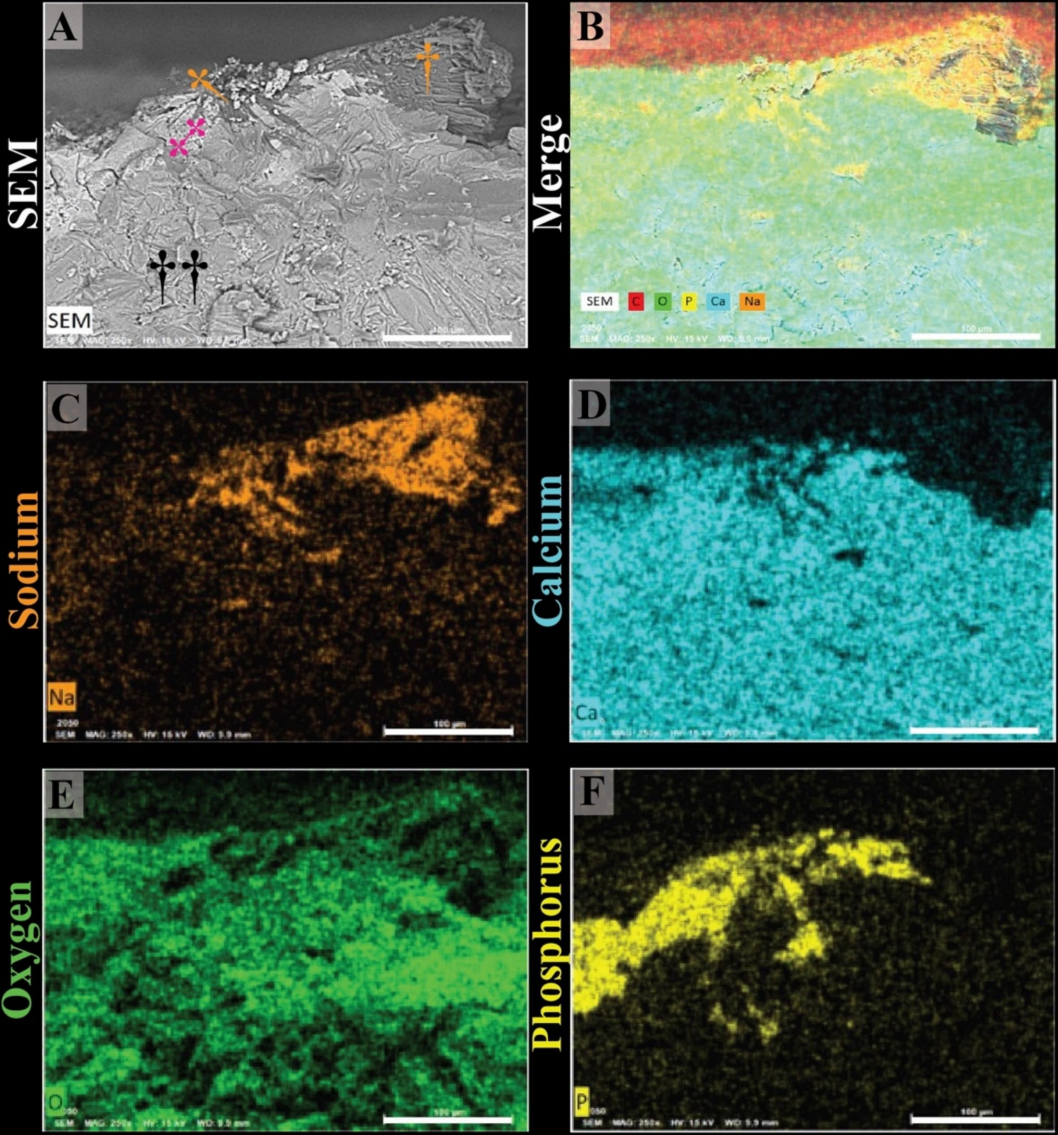
Energy dispersive spectroscopy (EDS) analysis of the RP stone from Patient 6 confirms the presence of MSU crystals within Randall’s plaque (RP). **A** SEM image highlighting the region of interest in the stone: MSU crystals (yellow daggers), apatite (magenta double dagger) and calcium oxalate monohydrate (black daggers). **B** Composite image (merge) from the microanalysis of the stone showing the elements C, O, P, Ca and Na. EDS analysis confirms that the needle-shaped crystals integrated within RP are MSU, with sodium **(C)** detected in these regions. Areas without MSU crystals exhibit apatite (CaP) particles, which are typical of RP, as shown in **(D)** and **(F)**

RP in a stone from patient 6 was also found to contain MSU crystals integrated with apatite within the plaque as shown in Fig. 4. Stereoscopic imaging revealed the internal structure RP after careful sectioning for SEM analysis (Fig. 4C, D and E). A micro CT reslice of the stone, corresponding to the approximate section of fragment 2, is shown in Fig. 4B and aligns with the section viewed under SEM. SEM images (Fig. 4D and E) demonstrate the presence of the needle-shaped MSU crystals with the RP, with a close-up view (Fig. 4E, inset from **D**) revealing detailed crystal structures, including both CaP and MSU crystals. EDS analysis of the RP stone from Patient 6 confirmed the presence of MSU crystals within RP (Fig. 5). MSU crystals within RP were confirmed by EDS detection of sodium (Na) in those regions (Fig. 5C). The areas without MSU crystals demonstrated CaP particles, typical of RP, as shown in Fig. 5D and F.

## Discussion

Our results demonstrate that stereoscopic microscopy, micro CT, SEM, and EDS together offer a robust method for analyzing the stone initiation regions of RP stones. Indeed, our investigation on RP stones confirms that interstitial plaque predominantly contains CaP apatite, which may begin within the basement membranes of the loops of Henle as previously reported [19, 20]. Additionally, our SEM and EDS studies uncovered needle-like crystals composed of MSU intercalated with CaP apatite within kidney papillae, that may indicate the presence of inflammatory mediators that could contribute to local inflammation and facilitate RP development.

The results in Fig. 1 (and those shown in Supplemental Figure S2) present SEM images of RP stones obtained from three different patients. Our micro CT reslice images confirmed the presence of RP and facilitated the identification of the plaque for sectioning. SEM analysis reveals that the mineralized renal tubules within RP stones exhibit a wide range of luminal sizes, from approximately 13 µm to 113 µm in diameter. It is plausible that mineralized structures with tubular diameters between 15 and 22 µm represent thin loops of Henle, whereas those measuring approximately 20-50 µm are likely to correspond to collecting duct structures [21, 22]. Moreover, mineralized tubular structures were observed with luminal diameters as large as 113 µm (Fig. 1F and Supplemental Figure S2 S), which may correspond to terminal collecting ducts or papillary ducts of Bellini [23, 22]. Tubular structures in RP were all confirmed to be positive for calcium and phosphorus by EDS analysis (Supplemental Figure S1; EDS data for all are not shown).

Of particular interest was the discovery of MSU crystals intercalated with CaP apatite within RP. Among the eighteen RP stones analyzed, only three contained sodium urate crystals, derived from two distinct patients (patient #5 and patient #6, Supplemental Table 1). Figure 4E clearly shows the intercalating crystals of apatite (magenta double crosses) and monosodium urate (yellow daggers). The SEM details of this sectioned RP stone show sodium urate crystals radiating outward toward the apatite plaque, as if serving as nucleation sites for apatite crystal deposition. In fact, our interpretation of this observation is that the MSU crystals likely precipitated in the papilla before the apatite, given urate’s lower solubility in alkaline environments [24–28]. We suspect that upon the initial breach of the papillary urothelium, tissue fluids maintain a relatively alkaline pH in the region of RP nucleation. This interpretation is consistent with the observations made by Sethmann et al. [14]. Under these conditions, sodium urate is more likely than CaP to precipitate, given that uric acid is approximately 100 times more concentrated in the urine than in the interstitial fluid. Uric acid may enter the region of RP initiation, convert to urate, and precipitate [29]. This structural finding suggests that sodium urate may provide a niche for the deposition of CaP apatite in RP.

The finding of MSU crystals in the subset of RP stones reflects a pathological state reminiscent of gouty joints in synovial fluid [30, 31]. Macrophages and neutrophils are known to phagocytose MSU crystals in the joints and kidneys, triggering a specific local inflammatory response that persists in gouty joints and gouty nephropathy, primarily via the NLRP3 inflammasome along with other inflammatory cytokines [32–36]. Gouty nephropathy or a specific local inflammatory environment may act as a precursor to the development of RP in patients with COM stones on RP.

Indeed, it has long been suggested that inflammation plays a key role in the pathogenesis of nephrolithiasis. For example, elevated serum levels of the inflammatory marker C-reactive protein (CRP) have been reported in patients with kidney stones [37–39]. Linking inflammation to kidney stones disease is critical, as it suggests a potential avenue for stratifying different subtypes within a given stone phenotype. Identifying specific inflammatory pathways in stone disease may facilitate the development of targeted anti-inflammatory therapies [10, 40]

In a recent study, Dejban et al. reported that stone formers displayed a higher density of pro-inflammatory macrophages (M1 macrophages) in kidney tissue compared to non-stone formers. Moreover, M1 macrophage density correlated positively with mineral deposition in the renal medulla of stone formers [10, 41]. Taguchi et al. also identified local inflammation of kidney papilla biopsies containing RP, with macrophages, neutrophils and plasma cells contributing to the inflammatory environment [42]. Witzmann et al. quantified 1059 unique proteins from CaOx kidney stone patient samples, revealing the complexity of the stone matrix proteome. The most common proteins identified were related to the immune response, inflammation, injury and tissue repair [43]. Similar immune, inflammatory and injury signatures have been identified by others in tissues and stones of CaOx stone formers [41, 44–46, 49].

Work by Daudon et al. have previously shown the relative prevalence of the main components of Randall’s plaques. The authors noted the presence of carbapatite (90.8%), amorphous carbonated calcium phosphate (4.6%), sodium hydrogen urate (3.5%) and uric acid (0.5%). Their study noted that two-thirds of the plaques containing sodium urate also contained carbapatite crystals [11, 12]. The variability in the types of crystals found in RP may favor distinct inflammatory responses in the papillae potentially involving the NLRP3 inflammasome and macrophage-mediated inflammation, similar to MSU crystals in gout [47–49]. More recently, Williams et al. demonstrated that biopsy specimens from the RP CaOx stone former phenotype are distinguished by the accumulation of CD68^+^ M1 macrophages, especially around the interstitial plaque regions [9, 49].

Our findings underscore the power of SEM and EDS combined with micro CT in elucidating early mineralization events in RP. We provide new insights into the sequence of mineral formation, demonstrating that MSU crystals precede CaP apatite in a subset of RP patients, potentially defining a distinct RP stone-former phenotype. The presence of needle-like sodium urate crystals intercalated with apatite in kidney papillae suggests that inflammatory and immune responses, potentially involving specific macrophage signatures, pathways and other immune mediators, play a critical role in the initiation and progression of RP.

## Conclusions

Integrating micro CT with SEM and EDS provides an invaluable approach for analyzing RP stones. The combination of these techniques has allowed us to identify novel structural features within RP that may be critical in early plaque and stone development. Further research to validate and explore the interplay between sodium urate and apatite crystallization could inform new therapeutic strategies for preventing or treating papillary stone disease.

## Supplementary Information

Below is the link to the electronic supplementary material.

Supplementary file1 (PDF 20208 kb)

## Data Availability

No datasets were generated or analysed during the current study.
