# Supplementary material for: Scanning electron microscopy and energy dispersive spectroscopy of Randall’s plaque stones: an unexpected finding of monosodium urate crystals
Source: Urolithiasis. 2025 Sep 13;53(1):175. doi: 10.1007/s00240-025-01842-w (PMC12433363; doi:10.1007/s00240-025-01842-w)
Supplement: Supplementary file 1 — Supplementary file1 (PDF 20208 kb) [file 240_2025_1842_MOESM1_ESM.pdf]

Supplemental Table 1. Patient data.

Supplemental Table 1

Clinical and surgical data on patients with Randall's plaque stones

	**Patient 1**	**Patient 2**	**Patient 3**	**Patient 4**	**Patient 5**	**Patient 6**	**Patient 7**	**Patient 8**	**Patient 9**
#RP stones analyzed	1	3	1	2	7	1	1	1	1
Age	49	50	64	37	57	—	—	—	—
Sex	M	M	M	M	M	M	M	M	M
Body mass index	26	30	30	27	33	—	—	—	—
Age at first stone	—	27	64	21	42	—	—	—	—
Hypertension	—	No	No	No	No	—	—	—	—
Diabetes	—	No	No	No	No	—	—	—	—
Surgery	Both	URS	URS	URS	Perc	—	—	—	—
Serum creatinine (mg/dL)	1.22	1.21	1.05	1.19	0.9	—	—	—	—
Serum calcium (mg/dL)	9.67	—	9.6	10.1	9.7	—	—	—	—
Serum uric acid (mg/dL)	6.77	—	—	7.6	—	—	—	—	—
24-hour urine volume (L)	2.9	2.2	3.8	2.1	2.32	—	—	—	—
24-hour urine pH	6	6.2	6.1	5.72	5.9	—	—	—	—
24-hour urine citrate (mg)	402	574	965	279.5	567	—	—	—	—
24-hour urine calcium (mg)	391	394	184	225	120	—	—	—	—
24-hour urine oxalate (mg)	51	30	34	30	29	—	—	—	—
24-hour urine sodium (mEq)	375	222	145	162	147	—	—	—	—
24-hour urine CaOx supersaturation	5.75	5.8	2.49	11.5	3.23	—			—
24-hour urine CaP supersaturation	1.37	2.18	0.4	0.42	0.36	—	—	—	—

URS, ureteroscopy. Perc, percutaneous nephrolithotomy, or Both, both ureteroscopy and percutaneous nephrolithotomy.

Supplemental Material

Supplemental Figure S1

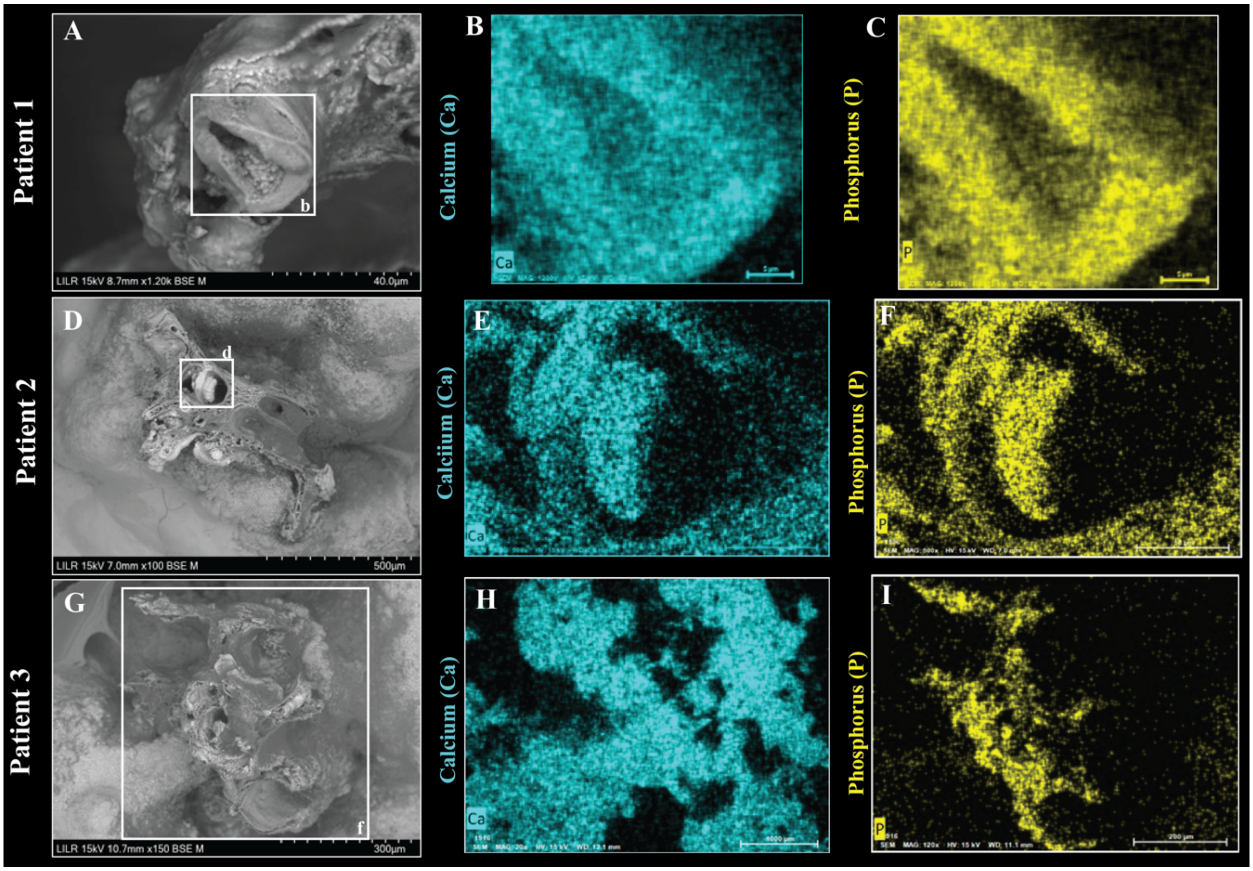

Supplemental Figure S1:
Confirmation of calcium phosphate by EDS in Randall's Plaque stones shown in main Figure 1.

Supplemental Figure S2

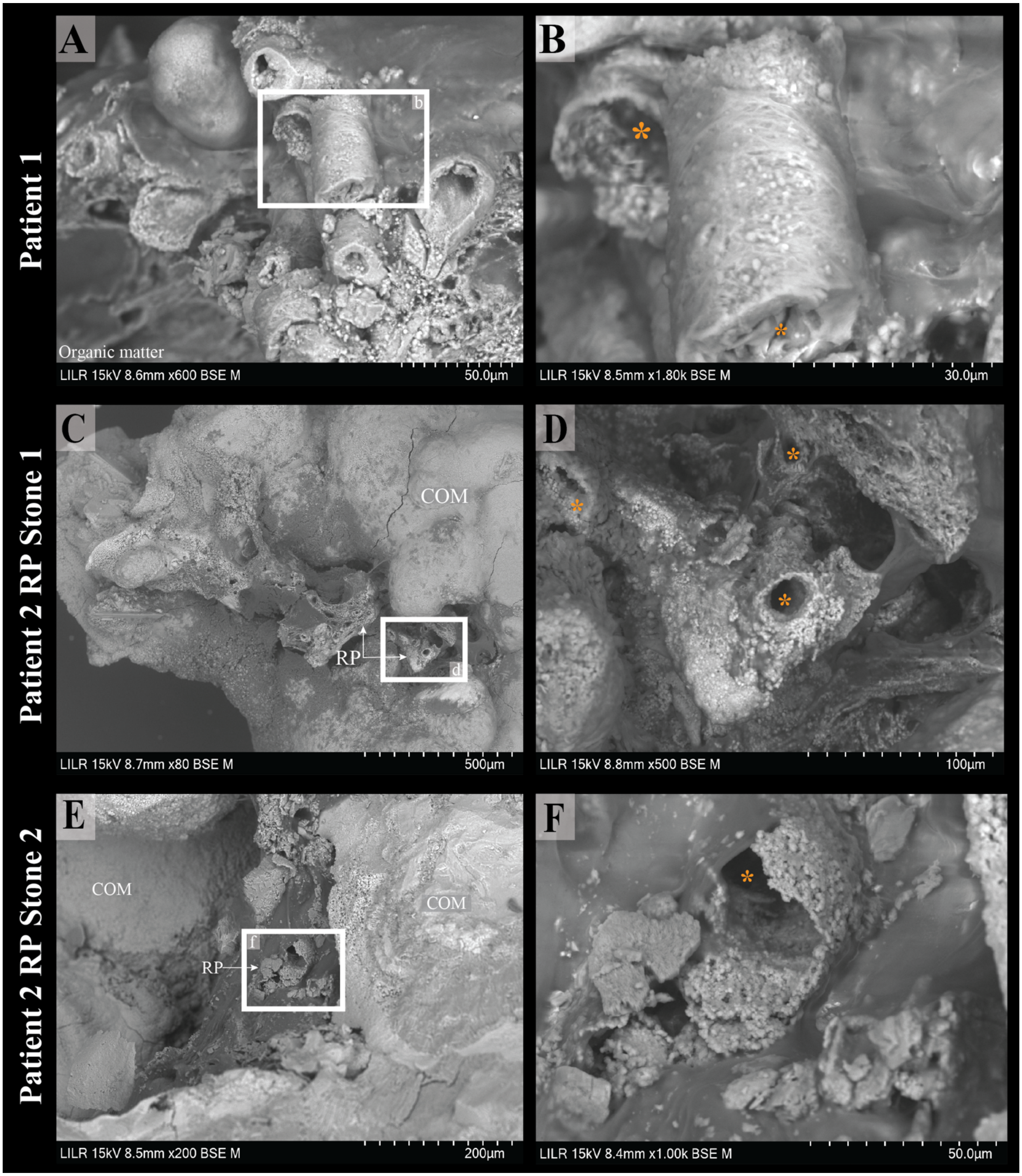

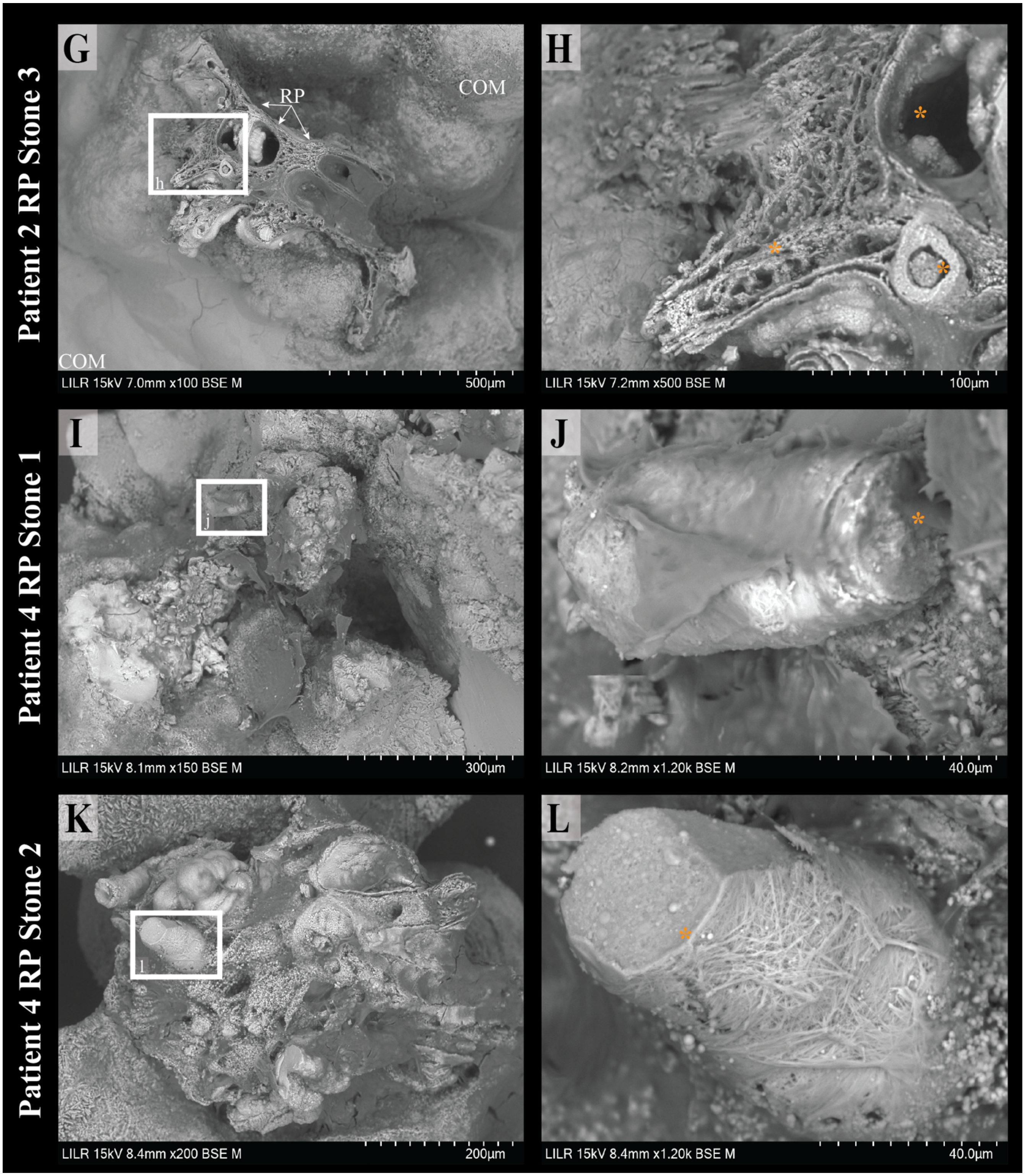
Patient 2 RP Stone 3
Patient 4 RP Stone 1
Patient 4 RP Stone 2
G
COM
RP
h
COM
LILR 15kV 7.0mm x100 BSE M
500µm
H
LILR 15kV 7.2mm x500 BSE M
100µm
I
j
LILR 15kV 8.1mm x150 BSE M
300µm
J
LILR 15kV 8.2mm x1.20k BSE M
40.0µm
K
l
LILR 15kV 8.4mm x200 BSE M
200µm
L
LILR 15kV 8.4mm x1.20k BSE M
40.0µm

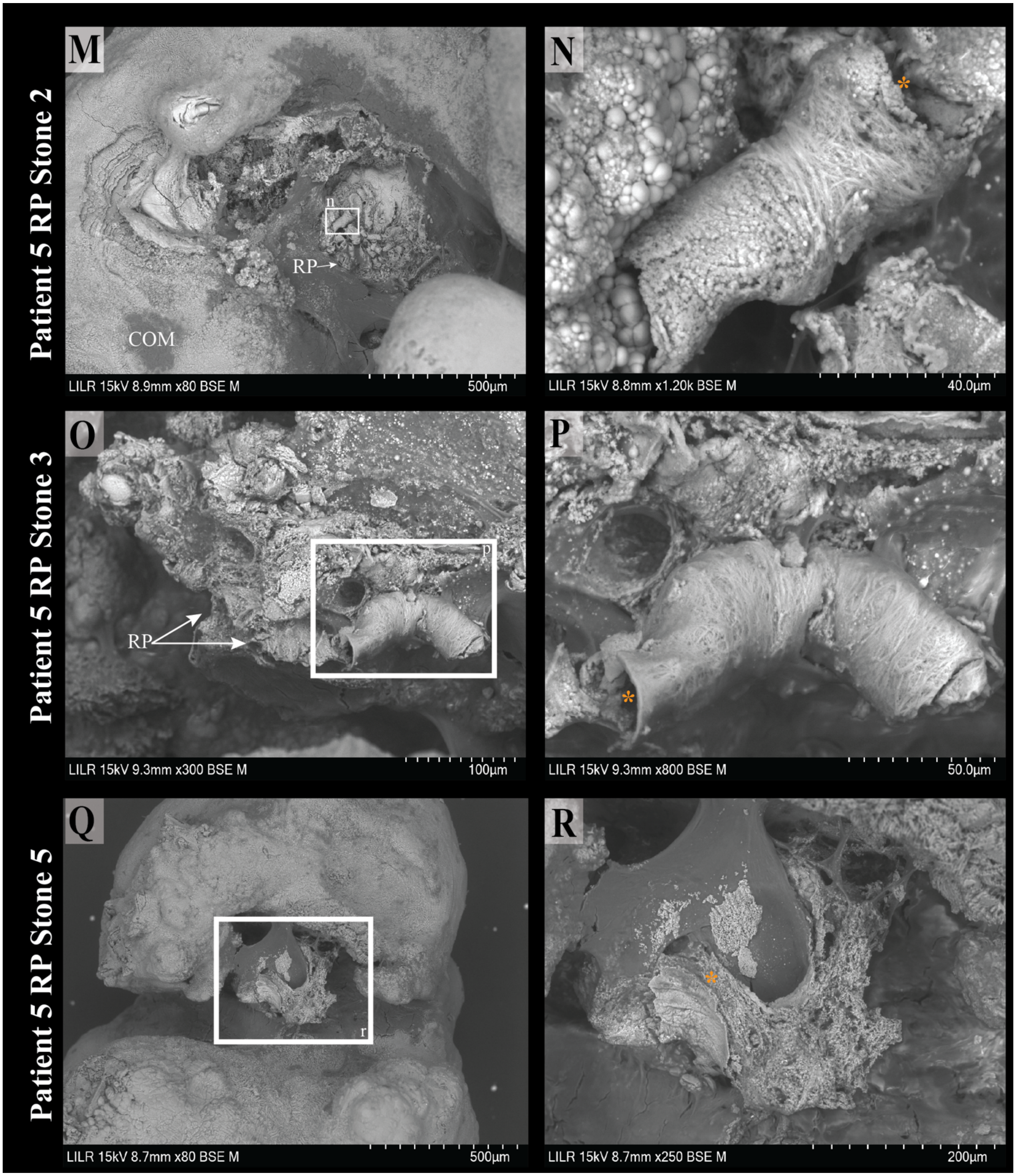

Patient 5 RP Stone 2
Patient 5 RP Stone 3
Patient 5 RP Stone 5
M
n
RP
COM
LILR 15kV 8.9mm x80 BSE M
500µm
N
*
LILR 15kV 8.8mm x1.20k BSE M
40.0µm
O
p
RP
LILR 15kV 9.3mm x300 BSE M
100µm
P
*
LILR 15kV 9.3mm x800 BSE M
50.0µm
Q
r
LILR 15kV 8.7mm x80 BSE M
500µm
R
*
LILR 15kV 8.7mm x250 BSE M
200µm

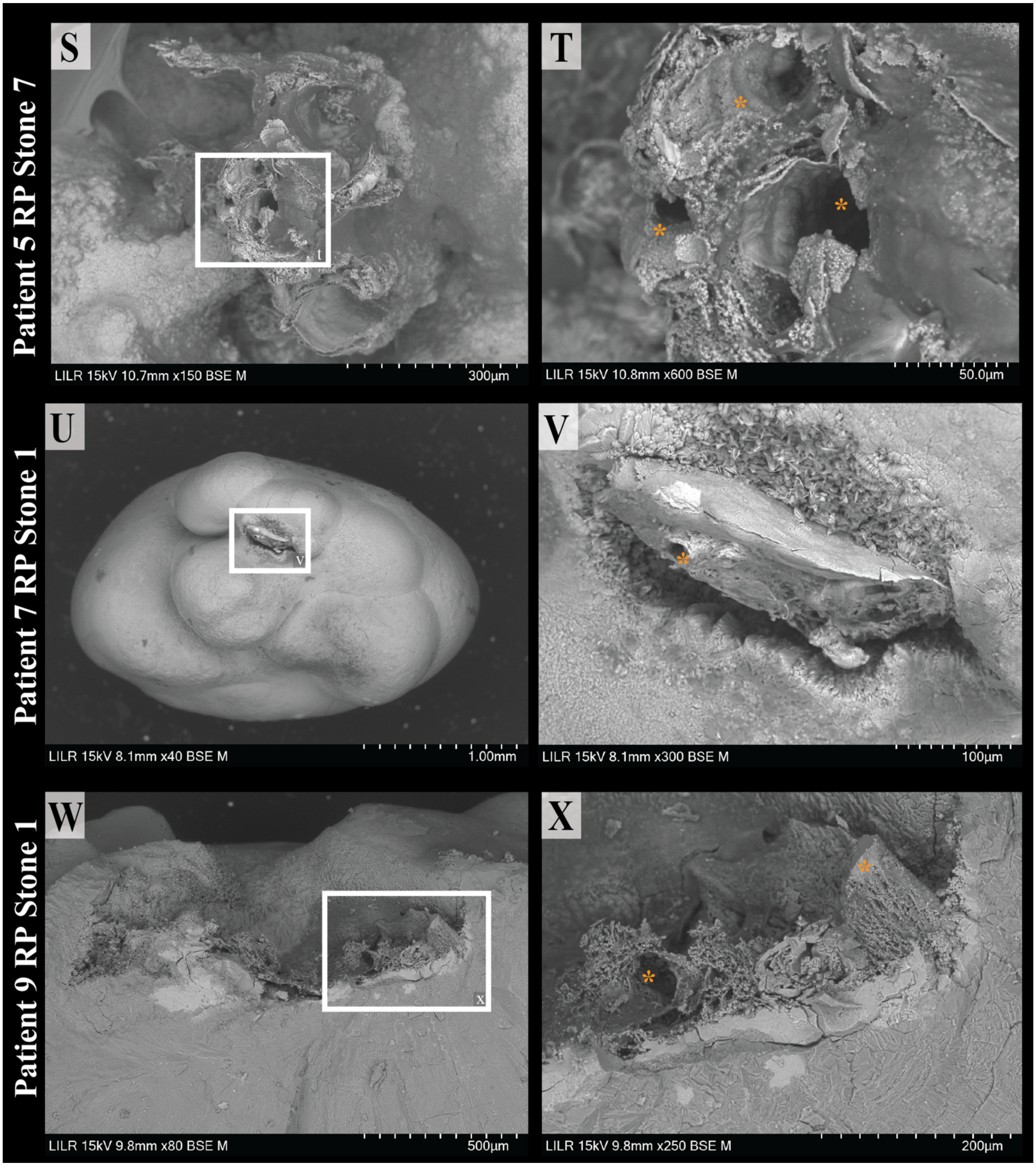

Supplemental Figure S2. RP stones showing at least one renal tubule, e.i., thin limb, collecting duct or papillary duct of Bellini. A yellow asterisk (*) can be found in the lumen of such structure in or on it.

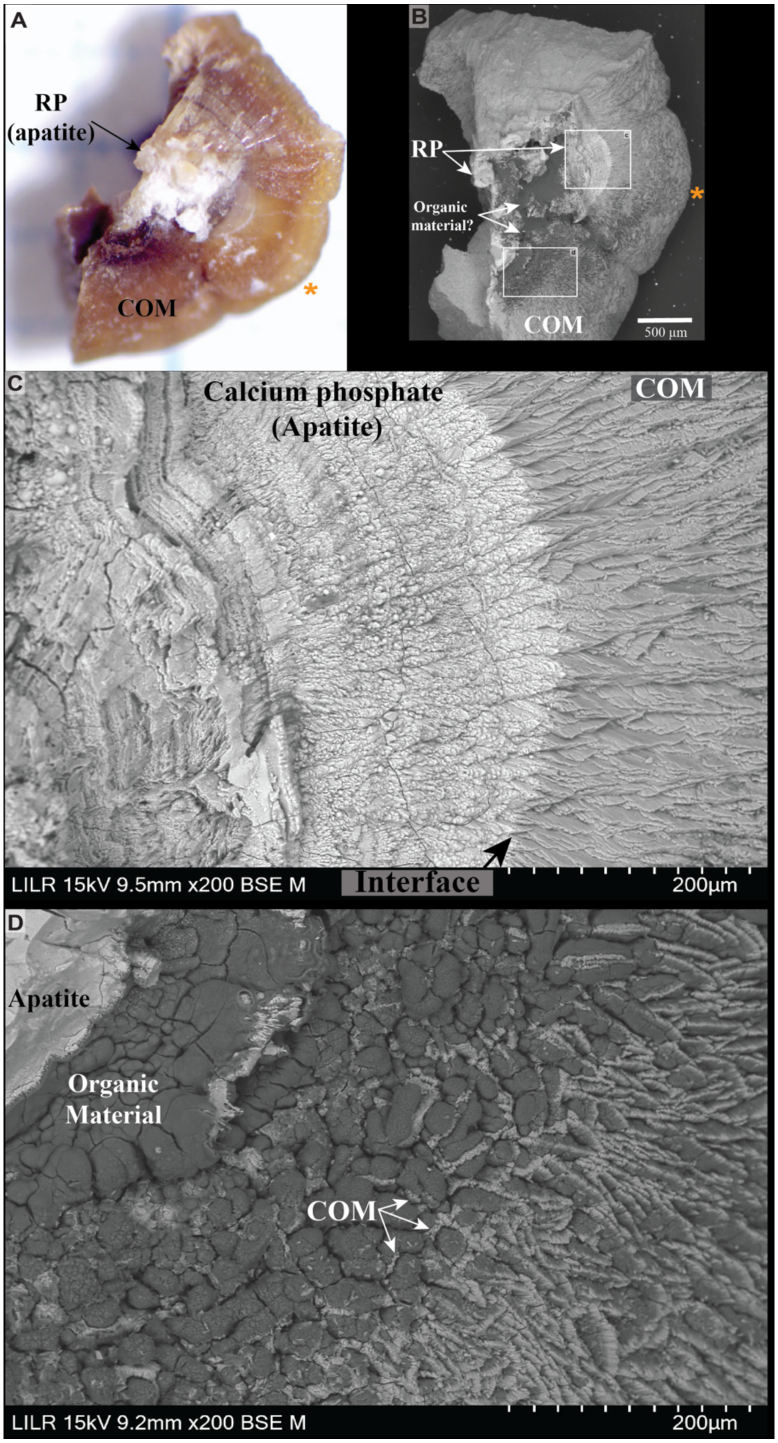

Supplemental Figure S3. A Stereoscopic image of a Randall's plaque (RP) stone from Patient 3, highlighting apatite regions with calcium oxalate monohydrate (COM) crystal overgrowth. **B**. Scanning electron microscopy (SEM) image of the same stone, with regions of interest indicated in insets c and d. **C**. Magnified view of inset c, showing the interface between apatite and COM crystals. **D**. Magnified view of inset d, depicting the interface region between apatite and what appears to be dried organic material, with COM crystals anchored to the organic matrix.

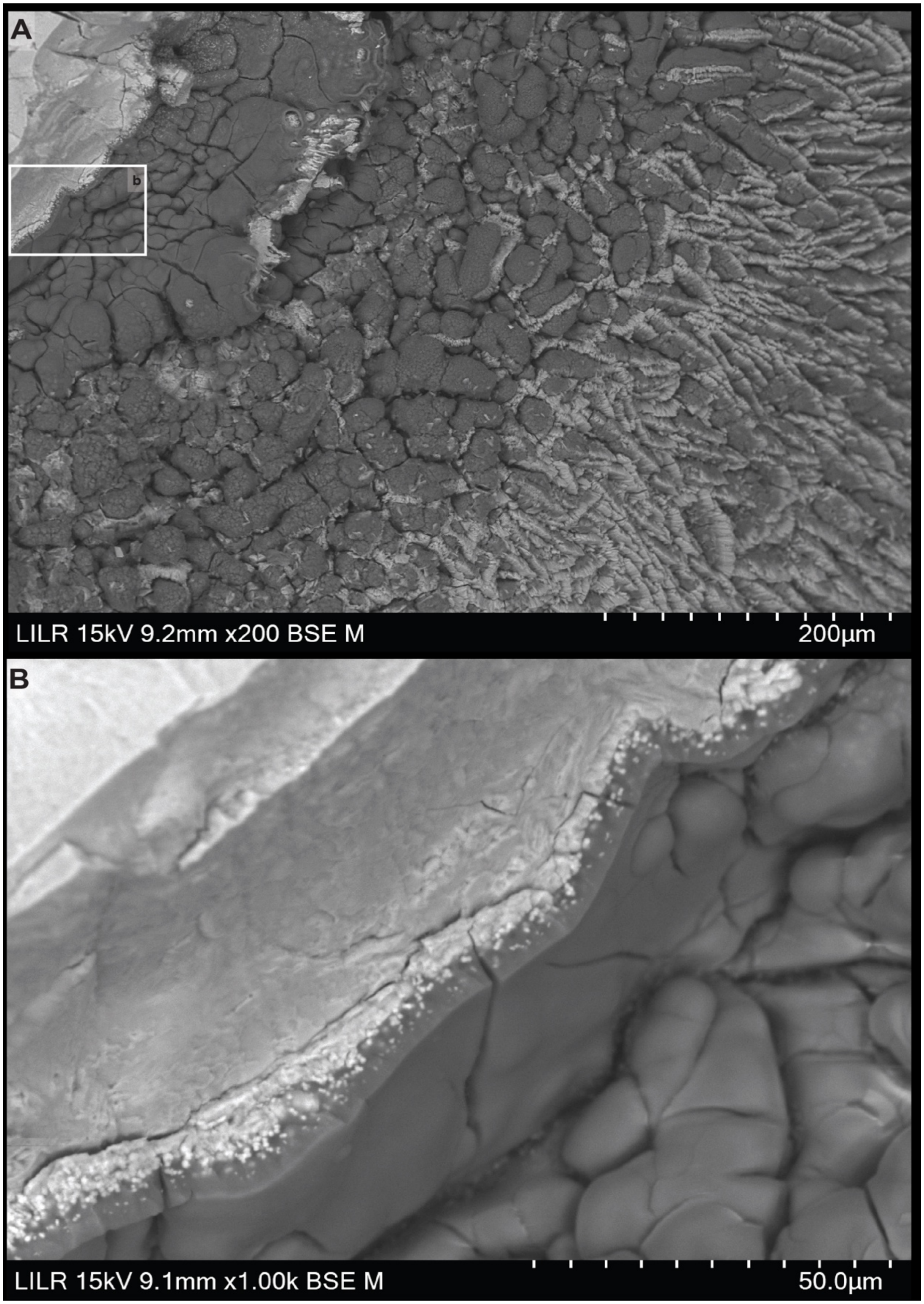

Supplemental Figure S4. A. RP stone from Patient 3, as shown in Supplemental Figure S3.**B.** This figure provides additional magnified views of the apatite-organic interface. Features at this interface shows a direct interaction between the mineral and organic components. Energy dispersive spectroscopy (EDS) data not shown.
